# Mechanisms and Model Diversity of Trade-Wind Shallow Cumulus Cloud Feedbacks: A Review

**DOI:** 10.1007/s10712-017-9418-2

**Published:** 2017-07-14

**Authors:** Jessica Vial, Sandrine Bony, Bjorn Stevens, Raphaela Vogel

**Affiliations:** 1 Laboratoire d’Océanographie et du Climat: Expérimentations et Approches Numériques (LOCEAN), Université Pierre et Marie Curie, Boîte 100 - 4, place Jussieu, 75252 Paris Cedex 05, France; 2 Laboratoire de Météorologie Dynamique (LMD), CNRS, Université Pierre et Marie Curie, Boîte 99 - 4, place Jussieu, 75252 Paris Cedex 05, France; 3 Max Planck Institute for Meteorology (MPI), Bundesstr 53, 20146 Hamburg, Germany

**Keywords:** Climate sensitivity, Global climate models, High-resolution models, Low-cloud feedbacks, Observations, Single-column models, Trade-wind shallow cumulus clouds

## Abstract

Shallow cumulus clouds in the trade-wind regions are at the heart of the long standing uncertainty in climate sensitivity estimates. In current climate models, cloud feedbacks are strongly influenced by cloud-base cloud amount in the trades. Therefore, understanding the key factors controlling cloudiness near cloud-base in shallow convective regimes has emerged as an important topic of investigation. We review physical understanding of these key controlling factors and discuss the value of the different approaches that have been developed so far, based on global and high-resolution model experimentations and process-oriented analyses across a range of models and for observations. The trade-wind cloud feedbacks appear to depend on two important aspects: (1) how cloudiness near cloud-base is controlled by the local interplay between turbulent, convective and radiative processes; (2) how these processes interact with their surrounding environment and are influenced by mesoscale organization. Our synthesis of studies that have explored these aspects suggests that the large diversity of model responses is related to fundamental differences in how the processes controlling trade cumulus operate in models, notably, whether they are parameterized or resolved. In models with parameterized convection, cloudiness near cloud-base is very sensitive to the vigor of convective mixing in response to changes in environmental conditions. This is in contrast with results from high-resolution models, which suggest that cloudiness near cloud-base is nearly invariant with warming and independent of large-scale environmental changes. Uncertainties are difficult to narrow using current observations, as the trade cumulus variability and its relation to large-scale environmental factors strongly depend on the time and/or spatial scales at which the mechanisms are evaluated. New opportunities for testing physical understanding of the factors controlling shallow cumulus cloud responses using observations and high-resolution modeling on large domains are discussed.

## Introduction

Over the past decades, marine boundary-layer clouds have emerged as a central issue for the projection and understanding of anthropogenic climate change. Because shallow cumulus and stratocumulus clouds cover large areas of the tropical and subtropical oceans, their response to global warming substantially impacts the Earth’s radiative budget. Climate models predict different low-level cloud responses to a warming climate, which results in a large dispersion in model-based estimates of climate sensitivity (Bony and Dufresne 2005; Webb et al. 2006). In the fifth Intergovernmental Panel on Climate Change (IPCC) assessment report (Boucher et al. 2013), global climate models (GCM) generally produce a positive low-level cloud feedback ranging between $-0.09$ and $0.63\, \hbox {W } \hbox {m}^{-2}\, \hbox {K}^{-1}$ (Boucher et al. 2013; Zelinka et al. 2016), which is primarily associated with a reduction in low-level cloud cover (Rieck et al. 2012; Bretherton et al. 2013; Brient and Bony 2013; Webb and Lock 2013; Zhang et al. 2013; Qu et al. 2014; Zelinka et al. 2016). Despite the apparent robustness in the sign of the low-cloud feedback among GCM (Zelinka et al. 2016), climate models suffer from important systematic biases in the present-day representation of marine boundary-layer clouds (e.g., Nuijens et al. 2015b) and physical mechanisms underlying cloud changes sometimes operate differently depending on whether they are parameterized (as in GCM) or largely resolved (as in high-resolution models). As a result, the confidence in the sign of the low-cloud feedback and therefore in the magnitude of climate sensitivity remains fairly low (Vial et al. 2016; Sherwood et al. 2014; Brient et al. 2015).

Although boundary-layer clouds are an integral part of a tightly coupled system, the structure and dynamics of these clouds appear to depend primarily on local processes acting at timescales that are much shorter than the large-scale dynamics (Neggers 2015a). These processes, which include turbulent and convective mixing, cloud radiative forcing and microphysics, remain unresolved at the typical grid size of standard GCM and thus have to be represented through parameterizations. Unfortunately parameterizations remain limited and model-based estimates of low-level cloud feedback and climate sensitivity depend on how cloud-related processes are parameterized (Zhang et al. 2013; Qu et al. 2014; Vial et al. 2016).

The confidence attributed to low-level cloud changes in a warming climate can only be improved by advancing the comprehension of the key processes that influence these clouds, ideally to the point where our understanding of factors controlling the cloud response can be tested against data (Klein and Hall 2015). Moreover, better process understanding of low-cloud changes contributes to the development and/or improvement in physical parameterizations and thus to the reduction in systematic model biases. Important contributions arose from the analysis of low-cloud feedbacks across a hierarchy of numerical models (Wyant et al. 2009; Brient and Bony 2012; Rieck et al. 2012; Blossey et al. 2013; Bretherton et al. 2013; Webb and Lock 2013; Zhang et al. 2013; Medeiros et al. 2015; Tan et al. 2017), through perturbed-physics model experimentations (Watanabe et al. 2012; Brient and Bony 2013; Tomassini et al. 2014; Zhao 2014; Webb et al. 2015; Vial et al. 2016) and by the use of process-oriented diagnostics in models and observations (e.g., Brient et al. 2015; Nuijens et al. 2015b).

This review aims to synthesize what is known about marine boundary-layer cloud feedbacks from observation- and model-based studies, focusing on the physical understanding of processes underlying the cloud response of fair-weather cumulus. As these clouds are most frequently observed in the trade-wind regions, they are often referred to as trade cumulus. Because, in climate models, trade cumulus cloud feedbacks are governed to a large extent by changes in cloud fraction near cloud-base in a warming climate (Brient and Bony 2013; Brient et al. 2015; Vial et al. 2016), a better understanding of the mechanisms that control cloudiness at lowest levels deserves particular attention. A number of studies have addressed this question over the past decades, including global and high-resolution modeling, and observational studies. But it appears that the cloud controlling factors on present-day timescales and the cloud feedback mechanisms in response to climate perturbations remain uncertain in this specific cloud regime.

Whereas inconsistencies in the response of stratocumulus to warming are thought to arise from differences in the balance of opposing feedback processes that are increasingly well understood (Bretherton 2015), the diversity of model responses of fair-weather cumulus appears to be more related to fundamental differences in how processes operate in models with parameterized, as opposed to resolved convection. Accordingly, we structured this review paper so as to emphasize two divergent interpretations of trade cumulus cloud feedbacks and mechanisms, as they emerged across the past decades, from the perspective of large-scale model parameterizations or from the perspective of Large-Eddy Simulations (LES). In Sect. 2, we discuss the first perspective, derived from the analysis of GCM. It considers changes in cloud-base cloud fraction as the main driver of trade cumulus cloud feedbacks and brings out the important role of parameterized convective mass fluxes in the diversity of model responses. In contrast, the interpretation of shallow cumulus cloud feedbacks at the process scale, based on theoretical considerations (Sect. 3) and LES (Sect. 4), suggests that cloud-base cloud fraction remains nearly invariant in response to climate change perturbations and that uncertainty in cumulus cloud feedbacks among LES is primarily driven by cloud changes near the trade inversion. In Sect. 5, we attempt to use a unified framework for GCM and LES results, to better interpret these contrasting views of trade cumulus cloud feedbacks and help consider the issue from a broader perspective. Finally, in Sect. 6 we discuss observational support for model-based trade-wind cumulus cloud mechanisms and consider opportunities for more discriminating observational tests.

## Interpreting Model Differences in Trade-Wind Cloud Responses to Warming in General Circulation Models

Because GCM are designed to simulate the evolution of the climate system at the global scale for hundreds of years, computational constraints limit the spatial resolution with which they can represent circulation systems. The effect of small-scale physical processes (such as turbulent and convective transports) on the resolved large-scale circulation must be parameterized. These parameterizations involve a large number of assumptions and numerical approximations that can affect the balance of the physical processes responsible for cloud formation and variability. This therefore causes large differences in cloud-topped boundary-layer structures among models (Brient et al. 2015; Nuijens et al. 2015b). Furthermore, at the time when parameterizations were developed for numerical weather prediction, the processes controlling low-level cloudiness were probably less of an interest as those clouds only represent a small contribution to the total cloud cover in many circulation regimes. Therefore, for the purpose of getting the total cloud cover right, parameterizations were tuned and harmonized to give a good representation of the present climate (e.g., Tiedtke 1989), which only indirectly constrains how cloud might respond to a changing climate.

### Boundary-Layer Moisture Budget

To better understand the behavior of the parameterized physics within GCM, we consider the budget equation of moisture, which in its simplest form (Eq. 1) describes the time rate of change of water vapor (*q*) as a function of source and sink terms, namely condensation (*c*) and evaporation (*e*), respectively:

1 $$\begin{aligned} \frac{D q}{D t} = c - e \end{aligned}$$

To solve this equation in a numerical model, we use its Eulerian form (Eq. 2), which then includes a local rate of change in *q* ($\partial q/\partial t$) and its evolution resulting from transport ($\mathbf {U} \cdot \nabla q$):

2 $$\begin{aligned} \frac{\partial q}{\partial t} + \mathbf {U} \cdot \nabla q = c - e \end{aligned}$$

To solve Eq. (2) in a large-scale model, the transport term is separated into two different types of transport: one by resolved fluid motions ($\mathbf {\overline{U}} \cdot \nabla \overline{q}$) and the other by unresolved fluid motions ($\partial (\overline{\omega 'q'}) / \partial p$, assuming horizontal homogeneity). In a GCM, the unresolved fluid motions are further broken down into two terms (convection and turbulence), so that to get the evolution of *q* requires different parameterized processes to interact with one another in a consistent way. Thus, the budget equation of moisture in a GCM can be written as:

3 $$\begin{aligned} \frac{\partial \overline{q}}{\partial t} = -\left[ \left( \mathbf {\overline{v}} \cdot \nabla \overline{q} \right) + \overline{\omega } \frac{\partial \overline{q}}{\partial p} \right] _{\mathrm {LS}} - \left. \frac{\partial (\overline{\omega 'q'})}{\partial p} \right| _\mathrm {turb} - \left. \frac{\partial (\overline{\omega 'q'})}{\partial p} \right| _\mathrm {conv} - (\overline{c} - \overline{e}) \end{aligned}$$

where physical parameterized processes affecting specific humidity and thus low-level clouds in subsidence regimes usually arise from separate schemes for turbulent diffusion in the boundary layer (turb), convection (conv) and net grid-scale condensation ($c - e$, which includes cloud formation, precipitation and evaporation and thus determines to a large extent the conversion to cloud water).

Large-scale low-level divergent winds in subsidence regimes act to export mass out of the boundary layer, which lowers the boundary layer. This is compensated by turbulent mixing that deepens and then dries the boundary layer as dry free tropospheric air is entrained into the boundary layer. In steady-state climates, this drying effect is compensated by moistening from the sum of the physical processes: the turbulence scheme is a source of moisture at lowest tropospheric levels, the convection scheme (when it is active) vertically transports moisture over the depth of the trade-wind layer from cloud-base up to overlying layers below the inversion or in the lower free troposphere and thus dries at levels near cloud-base (this transport is now commonly called *lower-tropospheric convective mixing* or *shallow convective mixing*), and the condensation scheme, which is the direct source of cloud water, is usually a sink term for the boundary-layer moisture budget.

Coordinated multi-model intercomparison studies such as those conducted by CFMIP (the Cloud Feedback Model Intercomparison Project; Webb et al. 2016) offer a way to sample model structural uncertainties for a given idealized framework and perturbation. The single-column model (SCM) intercomparison carried out as part of the CGILS (CFMIP-GASS Intercomparison of LES and SCM; Zhang et al. 2012, 2013; Blossey et al. 2013; Bretherton et al. 2013) project focused on marine boundary-layer clouds under idealized large-scale forcings representative of three different cloud regimes. This review focuses on those cases where cumulus convection plays a role in the coupling.

**Fig. 1:**
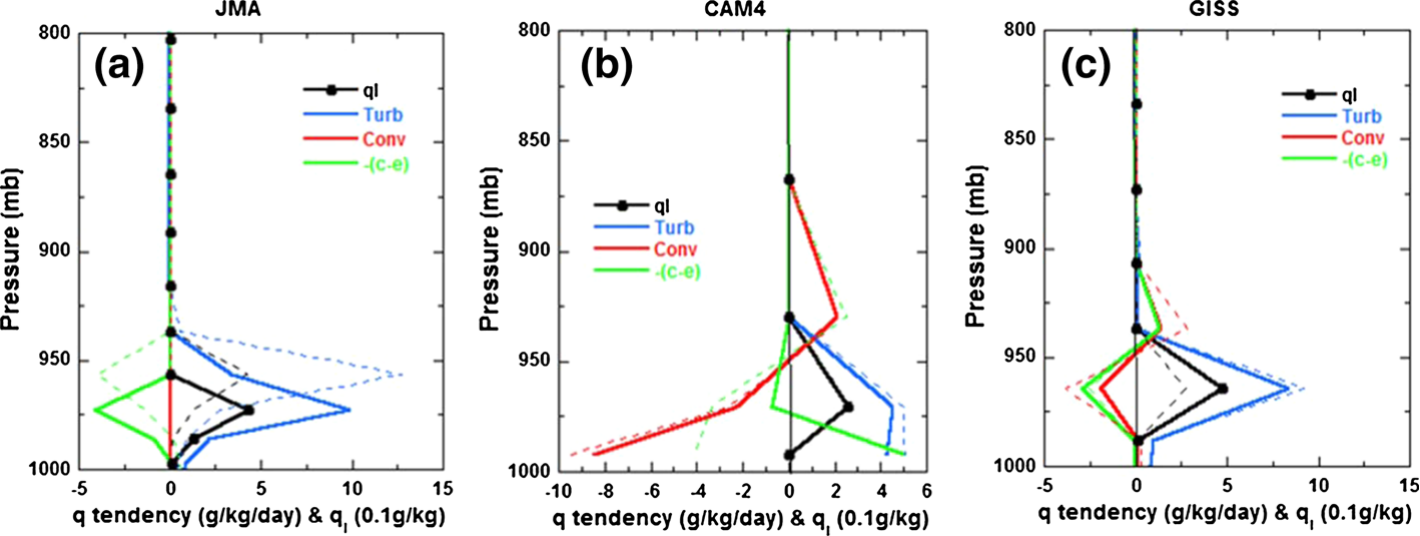
Physical tendencies of moisture (in $\hbox {g } \hbox {kg}^{-1} \hbox { day}^{-1}$) for decoupled stratocumulus (s11) in the present-day climate (*solid lines*) and in a warmer climate (*dash lines*): *turb* for the turbulence scheme, *conv* for the convection scheme, *c–e* for the net condensation scheme. *ql* represents the grid-averaged cloud liquid water ($0.1\hbox { g kg}^{-1}$, *black dotted line*). A sample of three SCM, having very different behaviors, is shown: **a** JMA (Japan Meteorological Agency), **b** CAM4 (Community Atmospheric Model Version 4), and **c** GISS (Goddard Institute for Space Studies). Note that although these profiles apply to decoupled stratocumulus, the sampled model diversity presented here remains relevant for shallow cumulus clouds. From Zhang et al. (2013)

**Fig. 2:**
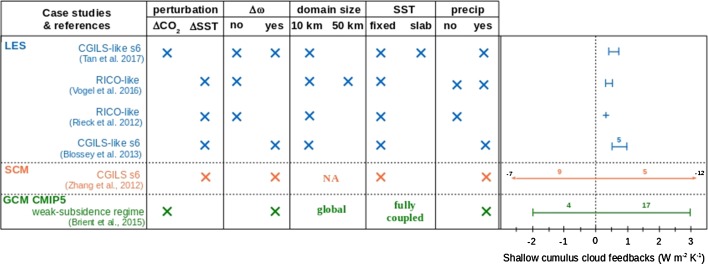
Synthesis of trade-wind shallow cumulus cloud feedback strength (in $\hbox {W m}^{-2}\hbox { K}^{-1}$) as simulated by different types of numerical models: LES (*blue*), SCM (*orange*) and CMIP5 GCM (*green*). For LES/SCM, different case studies are considered: CGILS-like s6 (Zhang et al. 2012; Blossey et al. 2013; Tan et al. 2017) and RICO-like (Rieck et al. 2012; Vogel et al. 2016). For each study, we provide, where applicable, information on the perturbed experiment used as surrogate for climate change ($\Delta SST$ or $\Delta CO_2$), if the large-scale subsidence ($\omega$) is perturbed or not, the domain size (small domain of ${\sim}10\hbox { km}$ or large domain of $\sim$50 km), if SST is prescribed (fixed SST) or interactive (the atmosphere is coupled to a slab ocean), and if precipitation is allowed or not. For multi-model studies, we indicate the number of models that simulate a positive or negative feedback (*colored numbers* on the *top* of the *arrow bars*). The *black numbers* at the extremities of the SCM *arrow bar* correspond to the minimum and maximum values of the simulated feedbacks

Different models balance their moisture budgets in regions of shallow cumulus in very different ways. This is illustrated in Fig. 1 (taken from a regime of mixed cumulus and stratocumulus convection), where differences in the convective mixing terms (tendencies) stand out when comparing how models maintain the present state and its response to warming. The ways in which these different balances influence the response to warming can be seen by considering what happens in a warmer climate. Because surface latent heat fluxes are expected to increase with warming (by about 2%/K—cf. Qu et al. 2015; Tan et al. 2017), we expect a larger turbulent moisture flux convergence in the cloud layer. In addition, the large-scale subsidence is reduced owing to the weakening of the tropical circulation. These two effects lead to increased cloud water (thicker and/or more abundant clouds). However, when convection plays a role, the enhanced moistening via turbulence and large-scale vertical advection is to a large extent compensated by enhanced drying from the export of condensate and the shallow convection (in a warming climate). If the rate of drying from the shallow convection is greater than the rate of moistening from turbulence and large-scale vertical advection, then we expect less condensation and less cloudiness, which would constitute a positive cloud feedback on the radiative forcing (as in Fig. 1c). Zhang et al. (2013)’s findings suggest that cloud feedbacks tend to be negative in models where parameterized convection is not playing an important role in balancing the moisture budget. The inter-model spread in this cloud regime for this SCM intercomparison is presented in Fig. 2 (in yellow). This large model diversity in shallow cumulus cloud feedbacks is primarily due to differences in cloud fraction changes at lowest atmospheric levels, where the effect of convective drying is the most important.

### The Role of Shallow Convective Mixing

In a warmer climate, the enhanced rate of drying by the shallow convection is similar to the *thermodynamic response* described by Rieck et al. (2012), Blossey et al. (2013) and Bretherton et al. (2013) on the basis of their analysis of LES results. More specifically, it was found that when just a surface (and/or atmospheric) warming is applied (while keeping the subsidence unchanged), the moisture gradient between the saturated air at surface and the drier free tropospheric air increases, yielding more efficient drying of the boundary layer by cloud top entrainment and/or vertical mixing by shallow convection (for a given entrainment/mixing rate). It is noteworthy as well that, in both LES and GCM, the presence of a stronger humidity gradient can also be interpreted as an enhanced subsidence drying (from an Eulerian point of view, which takes the equilibrium depth of the boundary layer fort granted); this provides an additional drying on top of the convective drying.

To better understand how convective mixing influences cloud amount, Vial et al. (2016) developed an analysis framework which allowed them to explore how changes in the convective mixing influence cloudiness in conditions reminiscent of trade cumulus convection. Using a single-column configuration of the Institut Pierre Simon Laplace (IPSL) model, they performed experiments using two different convective parameterization schemes. Their framework starts from the well-recognized result that the boundary-layer cloud fraction is mainly influenced by two antagonistic mechanisms: (1) the shallow convective mixing that dries the lower atmosphere and reduces the cloud fraction (Stevens 2007; Rieck et al. 2012; Zhang et al. 2013; Brient et al. 2015) and (2) the boundary-layer turbulent moistening (or latent heat flux) that enhances the cloud amount at low levels (Rieck et al. 2012; Webb and Lock 2013; Zhang et al. 2013; Brient et al. 2015). They thus expressed the sensitivity of the boundary-layer cloud fraction (${\mathrm {d}}f$) to a change in convective mixing (${\mathrm {d}}\mu$) and latent heat flux (*E*) as:

4 $$\begin{aligned} {\mathrm {d}}f&= {\mathcal {C}} {\mathrm {d}}\mu + {\mathcal {T}} {\mathrm {d}}E \end{aligned}$$

where the first term on the right-hand side describes the sensitivity of cloud fraction to convective (${\mathcal {C}}$) mixing, the second to turbulent (${\mathcal {T}}$) mixing. The model thus attempts to encapsulate the interplay between the two parameterizations used to model the transport of eddies as in Eq. (3). More specifically:

- ${\mathcal {C}}$ is the reduced cloud fraction when lower-tropospheric convective drying is enhanced under the effect of increased mixing (${\mathcal {C}} \equiv \left. \dfrac{\partial f}{\partial \mu }\right| _{E} < 0$)
- ${\mathcal {T}}$ is the increased cloud fraction when lower-tropospheric turbulent moistening is enhanced through increased latent heat flux (${\mathcal {T}} = \left. \dfrac{\partial f}{\partial E}\right| _{\mu } > 0$)

Using a series of sensitivity experiments, they showed that it was possible to linearly relate the surface latent heat fluxes to changes in the convective mixing (${\mathrm {d}}\mu$) and changes in the net boundary-layer cloud radiative effect (${\mathrm {d}}R$) as:

5 $$\begin{aligned} {\mathrm {d}}E&= \lambda {\mathrm {d}}\mu + \lambda _{\mathrm {r}} {\mathrm {d}}R \nonumber \\ {\mathrm {d}}E&= (\lambda + \alpha {\mathcal {C}} \lambda _{\mathrm {r}}) {\mathrm {d}}\mu \end{aligned}$$

where the variations in the net cloud radiative effect are essentially driven by the longwave cloud radiative cooling (R > 0 by convention) and linearly related to ${\mathrm {d}} f$, such as ${\mathrm {d}} R = \alpha {\mathrm {d}} f = \alpha {\mathcal {C}} {\mathrm {d}} \mu + \alpha {\mathcal {T}} {\mathrm {d}} E$ [see Vial et al. (2016) for more details on the simplifications that lead to the final form of Eq. (5)].

In Eq. (5), $\lambda$ and $\lambda _{\mathrm {r}}$ describe the two additional mechanisms that influence the latent heat flux, which can then modulate the sensitivity in boundary-layer cloud fraction to a change in convective mixing [see Vial et al. (2016) for more details on how $\lambda$ and $\lambda _{\mathrm {r}}$ are defined; here we just provide their physical description]:

- $\lambda$ is the increased latent heat flux through lower-tropospheric drying induced by the convective mixing ($\lambda > 0$), which damps the reduction in cloudiness.
- $\lambda _{\mathrm {r}}$ is the reduced latent heat flux as the lower troposphere stabilizes under the effect of reduced low-cloud radiative cooling ($\lambda _{\mathrm {r}} > 0$), which enhances the reduction in cloudiness.

By replacing ${\mathrm {d}}E$ into Eq. (4), the sensitivity of the boundary-layer cloud fraction to a change in convective mixing can be expressed as:

6 $$\begin{aligned} {\mathrm {d}}f&= \left[ {\mathcal {C}} + {\mathcal {T}}(\lambda + \alpha {\mathcal {C}} \lambda _{\mathrm {r}}) \right] {\mathrm {d}}\mu \end{aligned}$$

Using Eq. (6), the relative importance that the model assigns to the two processes (i.e., convective mixing and radiative cooling) can thus be measured by the magnitude of $\lambda$ and $\lambda _{\mathrm {r}}$. In the IPSL model, this depends to some extent on the closure of the convective parameterization. When this model uses a closure in stability (e.g., the convective available potential energy—CAPE), it exhibits a stronger sensitivity of low-level clouds to convective mixing in the present-day climate and a stronger low-level cloud feedback in response to surface warming, due to the prevailing coupling between latent heat flux and cloud radiative cooling ($\lambda _{\mathrm {r}}$). In contrast, when the IPSL model is run using a closure in subcloud moisture convergence, the coupling between latent heat flux and convective mixing ($\lambda$) dominates, which results in a lower sensitivity of cloudiness to convective mixing in the present-day climate and a weaker low-cloud feedback in a warming climate (Vial et al. 2016).

However, the closure of the convective parameterization is not the only assumption that can affect boundary-layer cloud feedbacks. In the CGILS SCM intercomparison (Zhang et al. 2013), two models having the same closure of the convective parameterization (CAPE) exhibit cloud feedbacks of opposite signs (the models differ also by entrainment/detrainment assumptions: one model includes lateral entrainment into the convective plumes, while the other does not). It is very challenging to determine how the different parameterizations fix the behavior of boundary-layer clouds, because they all are tightly connected to each other and with other parameterized and/or resolved processes (e.g., Vial et al. 2016). That said, this illustrates how different parameterization assumptions can affect the balance of the physical processes and boundary-layer cloud feedbacks, often in ways that were not considered when the schemes were designed. Following the Zhang et al. (2013) study, other process-oriented studies have then suggested that shallow convective mixing (and also more generally parameterized convection) appears as a leading source of inter-model spread in cloud feedbacks (Sherwood et al. 2014; Brient et al. 2015; Kamae et al. 2016; Vial et al. 2016).

Although convection is likely an important source of model diversity in the response of clouds in some regimes, the importance of other processes can also be important. This is shown for instance in experiments wherein convective cloud parameterizations are eliminated (Webb et al. 2015) and support the idea that the treatment of turbulence and cloud radiative effects also influences the evaporation and cloud amount (Vial et al. 2016).

Brient et al. (2015) have proposed another mechanism that could influence the change in convective mixing in a warmer climate, and thus the low-cloud feedback. Based on their analysis of the Coupled Model Intercomparison Project (CMIP5, Taylor et al. 2012) ensemble, they argue that increased near-surface stability in a warming climate weakens the sensible heat flux and limits the increase in latent heat flux. This in turn reduces the buoyancy flux and yields a shallowing of moisture mixing (due to weaker turbulent mixing) within the boundary layer and thus a shallowing of low-level clouds (with only subtle changes in cloud fraction). In their study, about half of the models favor this mechanism with respect to enhanced lower-tropospheric convective mixing as a result of increased surface evaporation. For these models, the low-cloud feedback is weaker (less positive). In contrast, in models where the changes in surface fluxes are more strongly related to changes in the trade-wind vertical humidity gradient (rather than near-surface stability), the moisture mixing deepens, yielding deeper clouds with a reduced cloud fraction at lowest levels and a more positive cloud feedback. In all models, the convective mixing is enhanced in a warmer climate, but models that simulate a low-cloud shallowing, with warming, are more influenced by the weakening of turbulent mixing (due to reduced surface sensible heat flux) and models that simulate a low-cloud deepening with warming are more influenced by the strengthening of convective mixing (due to increased surface evaporation).

A number of recent studies have used observations to evaluate which of the hypothesized mechanisms better describe the cloud response to changes in large-scale environmental conditions (e.g., Clement et al. 2009; Qu et al. 2014, 2015; Brient and Schneider 2016). These studies generally indicate that it might be the lower-troposphere mixing, although a complete demonstration of this mechanism using current observations remains difficult (this is a point we return to in Sect. 6).

The above discussion reflects our understanding of shallow cumulus cloud feedbacks and mechanisms from the perspective of large-scale model parameterizations of the trade-wind boundary layers (in GCM and SCM). In those models, cloudiness near cloud-base is the main driver of shallow cumulus cloud feedbacks and is strongly controlled by local interplays between turbulent, convective and radiative processes as a response to changes in large-scale environmental factors (e.g., surface/atmospheric temperature, vertical humidity gradient, subsidence). This is in contrast to what one finds in high-resolution modeling (e.g., LES), in which cloud fraction near cloud-base is nearly invariant with warming and independent of large-scale environmental factors that vary on long timescales. As a result, trade cumulus cloud feedbacks as simulated by LES are much smaller than usually simulated in GCM or SCM (Fig. 2). As discussed in the following sections, this contrasting behavior between GCM and LES appears to be related to the fact that large-scale climate models might lack cloud-base regulation processes between the cloud and subcloud layer, which in nature act to couple the turbulent fluxes in the subcloud layer with the convective fluxes within the cloud layer. In the following section, we provide the theoretical background used to rationalize the apparent constancy in trade-wind cloud fraction near cloud-base. Shallow cumulus cloud changes and mechanisms as simulated by LES are then reviewed in Sect. 4.

## A Mass Budget Perspective on Cloud-Base Cloud Fraction

Unlike what happens in most large-scale models, conceptual models of the layers of shallow convection [e.g., single-bulk layer models for the entirety of the trade-wind layer in Betts and Ridgway (1989) or subcloud layer models in Betts (1976)] emphasize how exchanges between the cloud and subcloud (well mixed) layers adjust the amount of mass in the subcloud layer so that its height remains close to the lifting condensation level (LCL). Such a process would imply that the humidity at cloud-base remains roughly constant. A closure of this form was used in early models of trade-wind cumulus (Albrecht et al. 1979; Betts and Ridgway 1989; Stevens 2006). By immediately adjusting the subcloud layer height to the LCL, these models essentially fix the humidity at cloud-base and by implication allow little room for cloudiness at cloud-base to vary with the cloud-base convective mass flux, *M*.

**Fig. 3:**
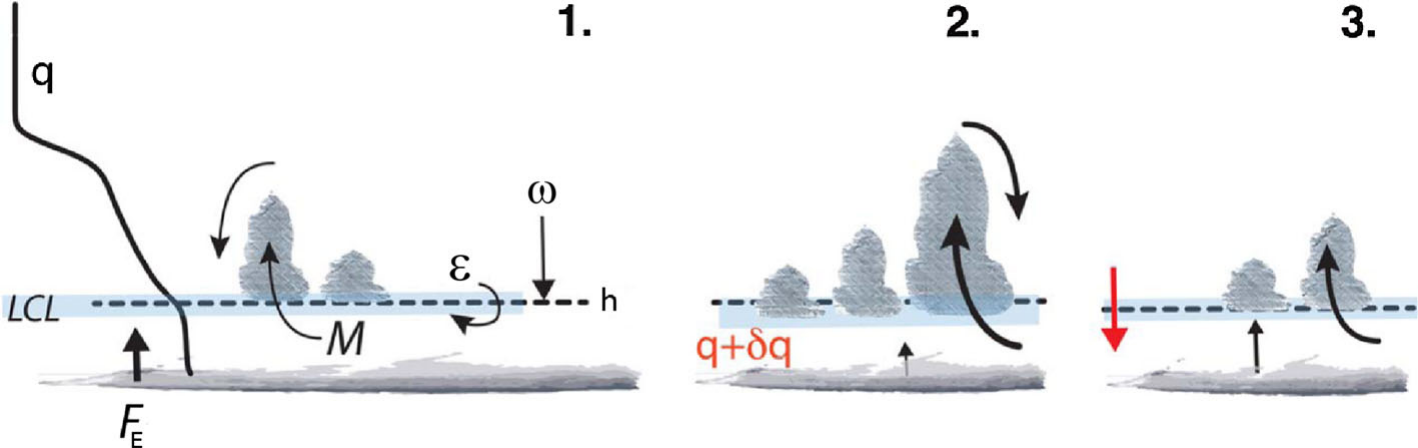
Illustration of the cumulus-valve mechanism. In (*1*) the system is at equilibrium, with a surface evaporation flux ($F_E$), a typical trade-wind humidity profile (*q*) roughly constant up to the top of the well-mixed layer at *h* (*dotted line*), clouds starting to form at the lifting condensation levels (LCL, represented by the *blue layer*) and the processes controlling the mass budget of the well-mixed layer (Eq. 7): the entrainment rate at *h* (${\mathcal {E}}$), the large-scale subsidence at *h* (*w*) and the convective mass flux (*M*). In (*2*) the humidity profile within the well-mixed layer is increased by *dq*. This reduces the surface evaporation, lowers the LCL, enhances the fraction of air parcels (including the cloud core fraction, $a_c$) and through the mass flux closure ($M = a_c w_c$) increases *M*. Larger *M* transports more moisture upward, which deepens trade-wind clouds and also yields more downward mixing of dry free tropospheric air to the mixed layer. In (*3*) a new equilibrium is reached whereby increased mixing has lowered the mixed-layer top close to the LCL again. Therefore, the fraction of saturated air parcels is reduced again (including $a_c$) and thus *M* is weakened. From Nuijens et al. (2015b)

The mass budget of the subcloud layer (illustrated in Fig. 3) provides the theoretical backdrop for this idea. Neglecting variations of density, $\rho ,$ within the shallow subcloud layer, the total mass (per unit area) of the layer can be written as $\rho h,$ where *h* is the depth of the layer, and

7 $$\begin{aligned} \rho \frac{{\mathrm {d}}h}{{\mathrm {d}}t} = \frac{1}{g} \left[ {\mathcal {E}} - \omega - M\right] \end{aligned}$$

This equation recognizes three source or sink terms: (1) the entrainment (${\mathcal {E}} > 0$) of air from the cloud layer into the subcloud layer, a mass source (of relatively dry and warm air); (2) the large-scale divergence of mass out of the layer, which by continuity is equal to the large-scale subsidence velocity ($\omega$) at *h*, for $\omega >0$ a mass sink; and (3) a convective mass flux ($M>0$), whereby cumulus convection evacuates mass out of the subcloud layer, a further sink. Assuming that the subcloud layer is well mixed, and neglecting downdrafts, only the entrainment term changes the properties of the subcloud layer air. The other source terms in Eq. 7 export mass with the same properties as the subcloud layer.

Neggers et al. (2006) adjust the subcloud layer height, *h*,  to the LCL through a closure on *M*. This cloud-base mass flux can be interpreted as being composed of the product of an effective area of convective active mass export out of the subcloud layer, $a_{{\mathrm {c}}}$, and the mean velocity of this export, $w_{{\mathrm {c}}},$ such that

8 $$\begin{aligned} M = \rho g \left( a_{{\mathrm {c}}} w_{{\mathrm {c}}} \right) . \end{aligned}$$

The Neggers et al. (2006) closure for *M* follows by parameterizing $w_{{\mathrm {c}}}$ as being proportional to the convective scale velocity, $w_{{\mathrm {c}}} \propto (h{\mathcal {B}})^{1/3}$ where ${\mathcal {B}}$ is the surface buoyancy flux and $a_{{\mathrm {c}}}$ as being proportional to the disequilibrium between the LCL and *h*,  or the humidity at the top of the subcloud layer. This means that, for a given $w_{{\mathrm {c}}};$ the larger the difference between *h* and the LCL, the larger is $a_{{\mathrm {c}}},$ and hence the larger is *M*.

To understand how this closure maintains *h* near the LCL, consider the perturbed scenario whereby the humidity of the subcloud layer is increased. As a result, the LCL will lower and the surface fluxes will decrease. The reduction in ${\mathcal {B}}$ has a small effect on $w_{{\mathrm {c}}}$ but this is more than offset by the increase in $a_{{\mathrm {c}}}$ arising from the larger difference between the LCL relative to *h*. As a result *M* is increased, thereby exporting more mass out of the subcloud layer and lowering *h*, bringing it closer to the LCL. This process is also illustrated schematically in panels 2 and 3 in Fig. 3. Note that the moistening of the subcloud layer also affects the entrainment term, both by changing the surface fluxes and slightly affecting the stability at the top of the subcloud layer, but for the purpose of our discussion these can be considered to be negligible. In practice, this mechanism can be thought of as a moisture convergence closure on *M*. It is sometimes called the *cumulus-valve mechanism* because the clouds act as a valve which helps maintain the top of the subcloud layer, *h*, close to the LCL and thus acts as a negative feedback of convection on the humidity, and presumably cloudiness, at the base of the cumulus layer.

**Fig. 4:**
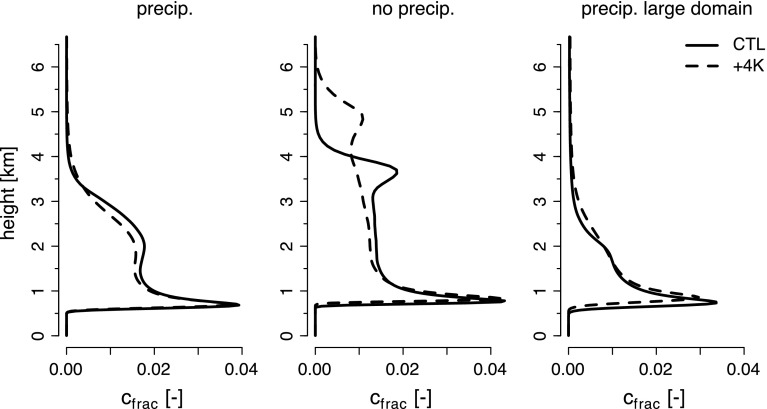
Domain-averaged vertical profiles of trade-wind shallow cumulus cloud fraction in LES (using the University of California Los Angeles—UCLA model) of precipitating clouds (*left*) and non-precipitating clouds (*middle*) over a small domain ($\sim 13 \hbox { km}$), and precipitating clouds over a larger domain ($\sim 50\hbox { km}$; *right panel*). The experimental setup is similar to that in Bellon and Stevens (2012) and consists of prescribed initial conditions and large-scale forcings (sea surface temperature, subsidence, radiative cooling and geostrophic wind) representative of the trade-wind regions. Results are shown for the present-day climate (*solid*) and as a response to a uniform warming of $+4$K at constant relative humidity (*dashed*). Figure adapted from Vogel et al. (2016)

The cumulus-valve mechanism has been evaluated at a specific shallow cumulus location but also in an tropical climate model with full interaction with the large-scale flow. Neggers et al. (2006) argue that the cloud fractions that result from the implementation of this closure are consistent with what is known about the climatology of shallow cumulus clouds from observations. A close inspection of their results shows that $a_{{\mathrm {c}}}$ indeed increases with *M*,  which implies a relatively moister subcloud layer, as air-moves though the trades over warmer waters. This in contrast to what one finds in parameterizations used in many climate models, whereby increasing *M*,  without adjusting *h* (which given the coarseness of the Eulerian coordinate in most parameterizations is hardly possible) dries, instead of shoals, the subcloud layer. This happens because, by not resolving variations in *h*, any convective mass out of the layer has to (by definition) be compensated by a flux of mass in (i.e., an implicit entrainment). Therefore, the GCM parameterizations effectively are increasing ${\mathcal {E}}$ to compensate for an increase in *M*; increased entrainment dries and warms the subcloud layer. A more careful accounting for the terms influencing the boundary-layer mass budget would (in the absence of a downdraft mass flux) not imply a subcloud layer drying, but rather a shoaling.

The above discussion illustrates how, when it comes to the humidity of the subcloud layer, old debates regarding closures for the convective mass flux have, it seems, unintended implications. In particular, the idea of the cumulus valve raises the question as to whether the strongly negative coupling between low-level cloudiness and convective mixing in many climate models (as shown in Sherwood et al. 2014; Brient et al. 2015; Vial et al. 2016; Kamae et al. 2016) may be a consequence of parameterizing the convective mass flux in a manner that does not sufficiently account for its link to the mass budget of the subcloud layer. Based on these ideas, and (as discussed in the following sections) the support they receive from measurements and large-eddy simulations, it is tempting to argue that many climate models generate cloud-base cloud fractions that are overly, or even wrongly, sensitive to the magnitude of the cumulus mass flux. In the case of the measurements, the lack of observations of key terms, such as the mass flux, hinders a conclusive interpretation using this framework (Bony et al. in revision). Evidence from LES presumes that the relative humidity at the top of the subcloud layer is the best determinant of cloud amount at cloud-base, and that LES—whose predictions of cloud-base cloud amount have not been critically evaluated against data (see Bony et al. in revision)—is a good surrogate for nature.

## High-Resolution Simulation of Shallow Cumulus Cloud Changes and Mechanisms

Unlike in climate models, where cloudiness near cloud-base is strongly controlled by convective and turbulent parameterizations as a response to changes in the large-scale environment (such as subsidence, surface temperature and vertical gradient of humidity—see Sect. 2), large-eddy simulation aims to explicitly resolve these convective and turbulent processes. Until quite recently computational restrictions only permitted LES over relatively small domains, which then required the parameterization of larger scale processes, usually by assuming that they can be specified independently of how turbulent and convective processes themselves develop. LES over larger domains are increasingly relaxing this assumption. Here we review what we know about shallow cumulus from LES, and whether LES is indeed doing a good job at capturing the observed vertical distribution and variability of shallow cumulus cloudiness. In so doing, we evaluate to what extent we can reject the strong cloud-base response to warming seen in many climate models, or at least what observations would be required to improve confidence in one or the other hypothesis.

### Trade-Wind Shallow Cumulus Cloud Response to Warming in LES

Overall, LES studies exhibit very small changes in cloudiness near cloud-base in response to surface and/or atmospheric warming. This suggests that the cumulus-valve mechanism (Sect. 3) may robustly constrain cloudiness at cloud-base in response to strong climate change perturbations (up to 8 K surface and atmospheric warming in Rieck et al. 2012). On the other hand, and unlike current climate models, LES models show that cloud changes near the inversion are the primary contributor to the total change in cloud cover (Rieck et al. 2012; Blossey et al. 2013; Vogel et al. 2016). The corresponding changes in cloud radiative effects appear robustly positive among LES studies, but much smaller than changes routinely simulated in global or single-column models (Fig. 2).

**Fig. 5:**
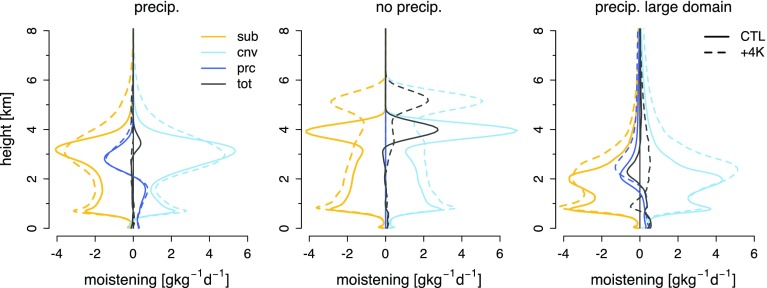
Domain-averaged vertical profiles of moisture tendencies: *sub* for the large-scale subsidence (*orange*), *cnv* for convection (diffusive and advective processes—in *cyan*), *prc* for precipitation (*blue*) and *tot* for the total moisture tendency (*black*). From left to right is for precipitating and non-precipitating simulations on a small domain ($\sim 13\hbox { km}$), and the precipitating simulation over a larger domain ($\sim 50\hbox { km}$). The same experimental setup as for Fig. 4 is used. Results are shown for the present-day climate (*solid*) and as a response to a uniform warming of $+4$K at constant relative humidity (*dashed*). Figure adapted from Vogel et al. (2016)

Cloud changes in a warming climate along with moistening tendencies in LES are illustrated in Figs. 4 and 5 (taken from Vogel et al. 2016).

In the absence of mesoscale organization and precipitation, the response of trade-wind cumulus to warming, as represented by LES, can be understood through simple bulk arguments (Rieck et al. 2012; Vogel et al. 2016). In a warmer climate, larger absolute humidity gradients imply that for a boundary layer of the same depth, which thus has the same rate of deepening to balance an assumed constant subsidence, the entrainment drying is larger. Stationarity implies a drier boundary layer so as to induce a sufficiently large moisture flux to balance this increased rate of entrainment drying (Fig. 5). But a larger moisture flux also implies deeper mixing and more drying, enhancing these effects further so that the equilibrium in a warmer atmosphere evolves to a deeper and drier cloud layer, with a reduction in cloudiness above ${\sim }1.5\, \hbox {km}$ (Fig. 4).

This is a typical view of shallow cloud feedbacks, which is similar to the thermodynamic mechanism for stratocumulus cloud reduction reviewed in Bretherton (2015), and similar to the thermodynamic response in many climate models (Sect. 2). However, the above arguments neglect precipitation, which introduces a new process in the balance of the water budget. Precipitation also affects the assumed structure of the boundary layer and the spatial organization (e.g., Seifert and Heus 2013). Motivated by these findings, Vogel et al. (2016) performed LES experiments to study the response of trade-wind cumulus clouds to warming for non-precipitating and precipitating shallow cumulus clouds. They also performed simulations on a large domain of about $50 \times 50\, \hbox {km}^{2}$ to better understand the role of organization. How these processes change our view of the balances determining cloudiness in the trade-wind layer are discussed below.

#### The Role of Precipitation

Studies of precipitating shallow cumulus (e.g., Blossey et al. 2013; Bretherton et al. 2013; Vogel et al. 2016) suggest that the main effect of precipitation is to restrain the deepening of the trade-wind layer, as explained in Stevens and Seifert (2008). Secondary effects arise from changes in the inversion and subcloud layer. With more precipitation, the cloud layer is more stable, but the inversion layer is less stable, so that clouds tend to detrain more continuously, leading to less stratiform cloudiness at the top of the cloud layer (compare left and middle panels in Figs. 4, 5). In addition, evaporation of precipitation in the lower part of the cloud layer induces a moistening and cooling, which yields an increase in cloud fraction near cloud-base compared to non-precipitating simulations. In a related study, Seifert and Heus (2013) explored the response of clouds to precipitation amount, rather than the differences between precipitating and non-precipitating simulations. They found that increasing precipitation leads to a reduction in cloud fraction over the whole trade-wind layer, including at cloud-base. Not withstanding that many of the responses to precipitation make physical sense, the magnitudes of the changes are not straightforward to assess. This is because, as a growing literature suggests, these are sensitive to the details of how the simulations are set up, ranging from the choice of microphysical schemes (Bretherton et al. 2013; Seifert and Heus 2013) to the effects of mean wind and resolution (Stevens and Seifert 2008; Matheou et al. 2011; Seifert and Heus 2013)—this point is further discussed in Sect. 4.2.

For precipitating layers, the response to warming is complicated by what are, at times, very strong changes in precipitation. For instance, in the warmer climate state of Vogel et al. (2016), increased surface fluxes with warming lead to congestus clouds developing with tops up 7 km. These dramatically change the structure of the boundary layer, weakening the inversion associated with more trade-wind-like clouds and limiting stratiform cloud formation. More compensating subsidence also leads to a shallowing and drying of the cloud layer, reducing cloud amount near cloud-base (Fig. 4).

#### The Role of Organization

Larger domain simulations (${\sim }50$ kilometers as in Seifert and Heus 2013; Vogel et al. 2016) allow shallow convection to organize in clusters of variable depth (depending on the domain vertical extension). The reasons for this organization are still being debated, but phenomenologically it shares similarly with convective self-aggregation as seen in simulations of deep convection (Wing et al. 2017). Clouds organized in clusters tend to produce larger amounts of precipitation, which generates evaporative downdrafts and initiates cold pools that spread out and trigger new convective cells at the cold pool boundary, where subsequent shallow cumulus clouds form. Because most of the precipitation remains concentrated in the convective clusters that populate the moist regions of the domain (e.g., in Vogel et al. 2016), evaporation of precipitation is reduced although the cloud layer is overall drier. These processes can also influence the response of clouds to warming.

The greater precipitation efficiency that accompanies mesoscale organization leads to a more stable and drier trade-wind layer. In addition, and with the help of compensating subsidence in the drier area, this effect keeps the trade-wind clouds in the rest of the domain shallow. Therefore, in the presence of organized convection, the trade-wind boundary layer is drier and more stable, and trade-wind cumulus clouds are shallower, compared to when shallow cumulus clouds are more randomly distributed in space (in smaller domain simulations). In a warming climate, upward convective transport of moisture strengthens in the large domain simulations, comparable to the small domain simulations (Fig. 5). Because the amount of deep cloud clusters is enhanced with warming, precipitation, as mentioned above, increases much more strongly with warming than in the small domain simulations (Vogel et al. 2016). Thereby, the drying due to precipitation tends to replace the drying due to large-scale subsidence, which cannot efficiently balance the enhanced convective moistening in the presence of a few deep cloud clusters and an otherwise very shallow and dry trade-wind layer (Vogel et al. 2016). As a result, different changes in cloud fraction and vertical distribution occur in the larger domain: clouds become deeper with a reduced cloud fraction near cloud-base—a feature that is not captured in the smaller domain (Fig. 4), yet is reminiscent of the dynamics seen in parameterizations (Sect. 2).

Does the reduction in cloud-base cloud fraction with warming imply that the presence of organized clusters in larger domain simulations can trigger mechanisms that overcome the internal cumulus-valve mechanism? Recently, Neggers (2015b) has shown that a mass flux framework that takes into account the spatial distribution of cumulus horizontal sizes can introduce interactions between convective plumes of different sizes (see also Seifert et al. 2015). In particular, if large cumulus clouds are more abundant than small clouds, the vertical convective fluxes tend to dry at low levels and transport moisture to higher levels. This low-level drying is compensated by the smaller cumulus plumes that detrain at levels where larger plumes remove moisture (Neggers 2015b). More study on the role of spatial organization and the influence of the cumulus size distribution on trade-wind shallow cumulus cloud variability and feedback appears important to have a more complete understanding of shallow cumulus cloud mechanisms. Note that the effect of spatial organization on larger domains and cumulus size distribution might be related to each other, as larger domains lead to organized clusters and therefore a larger proportion of cumulus with larger cloud-base area. The main point being that the constancy of cumulus base cloud fraction is not necessarily something that can be taken for granted.

### Robustness and Uncertainties of LES Studies

There is a tendency to view LES as surrogate of the truth, as able to fully represent the observed characteristics of the marine boundary layer. To some extent, this may be warranted by the robustness of simulated behavior across different LES. Simulated vertical distributions of cloud fraction and, to a slightly lesser extent, of projected cloud cover, tend to show relatively good agreement across different LES models in the Barbados Oceanographic and Meteorological EXperiment (BOMEX) and Rain In Cumulus over the Ocean (RICO) intercomparison cases of typical shallow trade-wind cumulus conditions (Siebesma et al. 2003; Van Zanten et al. 2011). An intercomparison case of the diurnal cycle of shallow cumulus over land also shows good model-to-model agreement (Brown et al. 2002). The cloud distributions of the above three intercomparison cases show a strong peak in cloud fraction at cloud-base, a rapid decrease in cloud fraction above cloud-base, and relatively small cloud fractions near the tops of cumulus clouds under the trade inversion. Total cloud cover ranges between about $13\,\pm 6\%$ for BOMEX (Siebesma et al. 2003) and $19 \,\pm \,9\%$ for RICO (Van Zanten et al. 2011), with the simulated cloud cover for RICO comparing favorably with corresponding lidar data. In simulations of an intermediate regime between stratocumulus and trade-wind cumulus, representative of the Atlantic Tradewind EXperiment (ATEX) field campaign and marked by a stronger inversion, the vertical distribution of cloud fraction has its maximum near the inversion instead of near cloud-base (Stevens et al. 2001). In this ATEX intercomparison case, there is more spread in simulated total cloud cover among the participating LES (total cloud cover ranges between 20 and 80% ($\hbox {mean }\pm 2\sigma$)), with the spread related to the representation of stratiform cloud amount under the inversion (Stevens et al. 2001). Also the CGILS intercomparison case of the response of shallow cumulus to climate change perturbations (location S6) shows the most apparent differences in the simulated cloud fraction profile near the top of the cloud layer under the trade inversion (Blossey et al. 2013). Whereas stratiform outflow layers are observed frequently at Barbados (Nuijens et al. 2014, 2015a), LES apparently have difficulties to properly simulate detrained layers of stratiform cloud. This difficulty is likely related to a poor representation of tight feedbacks between such outflow layers with radiation and subsidence, and to the fact that a very high vertical resolutions is necessary to resolve sharp inversions (Stevens et al. 2001). On the other hand, the range of cloud-base cloud fractions is quite consistent among the various intercomparison cases, with inter-model differences lying between 4.5 and 8% (Brown 1999; Stevens et al. 2001; Siebesma et al. 2003; Van Zanten et al. 2011; Blossey et al. 2013).

The comparison of the cumulative cloud fraction—the cumulative contributions to total cloud cover from the top down to the bottom of the cloud layer—estimated from LES and measured by a lidar indicates that the LES may not represent the full spectrum of cloud top height distributions present in nature (Figure 7 of Van Zanten et al. 2011). LES on large domains of ${\sim }50 \times 50\, \hbox {km}^2$ (about 16-times to 32-times larger than the domain sizes used for the intercomparison cases) can represent cloud populations with a wide range of cloud top heights, but cloud fractions in the upper cloud layer tend to be underestimated (Vogel et al. 2016). This underestimation is likely due to numerical diffusion, which is strongly related to the choice of advection scheme, the subgrid-scale model and the grid spacing. A thorough investigation of the impact of such model choices showed that cloud cover strongly decreased when a more dissipative monotone advection scheme was used instead of a centered differences scheme, or when a more dissipative subgrid formulation was used (relative decreases in cloud cover of up to 30%) (Matheou et al. 2011). Matheou et al. (2011) also find a relative decrease in cloud cover of up to 70% when the uniform horizontal and vertical grid spacing is increased from 20 m to 80 m. These results are in qualitative agreement with sensitivity studies presented in Stevens et al. (2001) and Siebesma et al. (2003) and show that one has to be careful when comparing absolute values of cloud cover between different LES studies, and between LES and observations. The strong decrease in cloud cover with larger grid spacing in Matheou et al. (2011) is partly due to reductions in cloudiness under the inversion, which cannot be resolved well at a vertical grid spacing of 80 m (see the liquid water specific humidities in their Figure 11). This again highlights that cloudiness near the top of shallow cumulus under the trade inversion is still poorly constrained by LES.

Studying how fields of shallow cumuli change in response to climate change perturbations and how they affect the planetary albedo and equilibrium climate sensitivity is also challenging using the current LES experimental setup. In this respect, LES yield an important limitation for climate studies, since they usually have to be run over small domains (10 to 50 kilometers) and therefore cannot realistically represent their variability under the wide range of conditions observed in nature, and especially their interactions with the large-scale circulation. For the same reason, LES have to be run over limited periods of time (a few days) and under simplified configurations (e.g., prescribed radiative cooling rate and sea surface temperature (SST)), and therefore all the process-scale interactions in the trade-wind layer and with the underlying ocean surface are not represented—for instance, the local interplay between cloud radiative forcing and turbulence as in Vial et al. (2016) and SST feedbacks on the trade-wind layer as in Tan et al. (2017).

Increasing computational resources now makes it possible to consider LES over larger domains, over larger timescales and under increasingly “realistic” configurations. For instance, simulations at 100 m resolution over the entire tropical Atlantic on timescales of months are now becoming possible with the ICOsahedral Non-hydrostatic (ICON) atmospheric model (Zängl et al. 2015; Heinze et al. 2016). Combined with observations of the planned field campaign $\hbox {EUREC}^4\hbox {A}$ (Elucidating the Role of Cloud-Circulation Coupling in Climate) over this region (discussed next), these simulations will provide new opportunities to study in more details the key factors controlling the cloud responses to warming, including the interactions between the trade-wind boundary-layer processes and the large-scale mesoscale organization.

## Connecting LES and GCM Interpretations of Shallow Cumulus Cloud Feedback Mechanisms

**Fig. 6:**
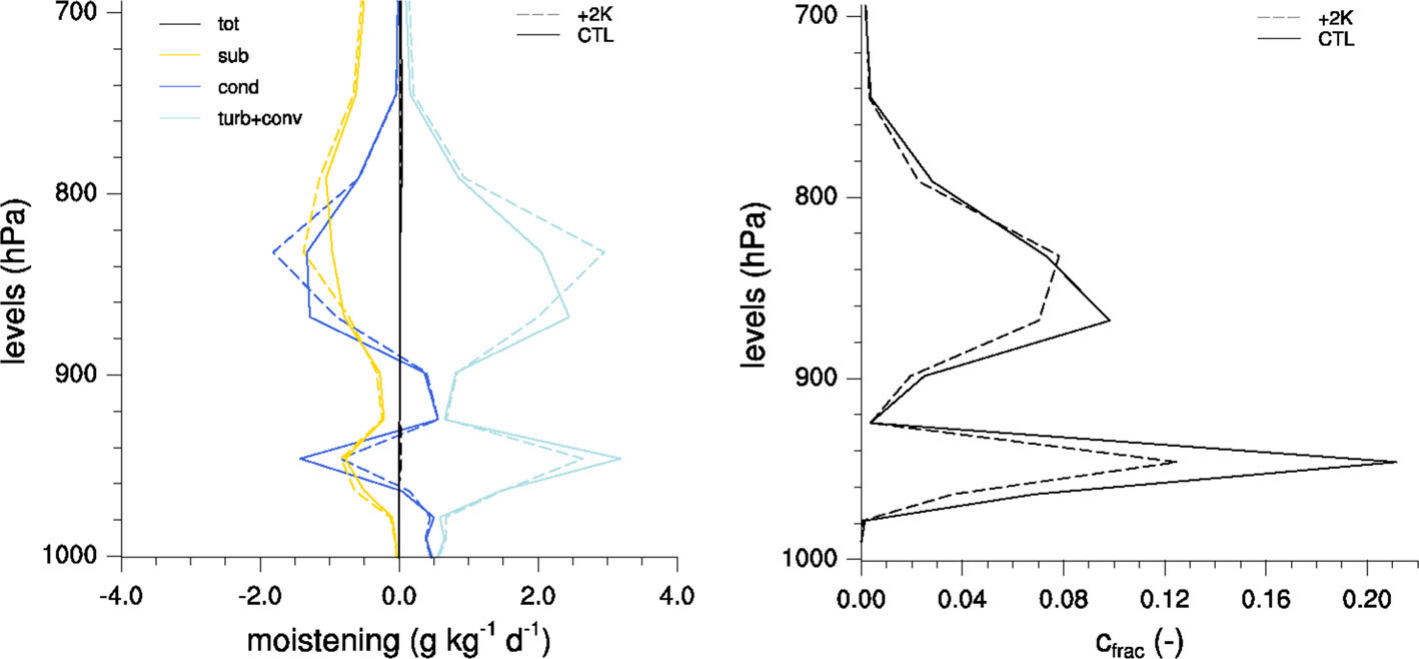
Vertical distribution of moisture tendencies (*left*) and cloud fraction (*right*) for a SCM experiment under CGILS framework (shallow cumulus regime, s6) using the IPSL-CM5A-LR GCM. Moisture tendencies include: turbulence and convection (*cyan*), grid-average net condensation (*blue*, which includes cloud formation, precipitation and evaporation), large-scale subsidence (*orange*) and the sum of all moisture tendencies (*black*). Results are shown for the present-day climate (*solid*) and as a response to a surface warming of $+2$K (*dash*). Adapted from Vial et al. (2016)

In order to better compare low-cloud changes and mechanisms between LES and GCM (or similarly SCM), a common interpretation framework is needed. Unlike in GCM and SCM, where turbulence and convection are usually represented by separate parameterization schemes (cf. Fig. 1), in LES these processes are part of a continuous spectrum of motions ranging from turbulent eddies (diffusive processes) to convective vertical drafts (advective processes). Convection is generally represented by the advective and diffusive flux divergence of the resolved and subgrid-scale flow and acts as a source of moisture over the whole trade-wind boundary layer (cf. Fig. 5). By adding the turbulent and convective tendencies in a GCM or SCM, tendency profiles comparable to LES can be generated (see Fig. 6 for a SCM example). In both LES and GCM, these turbulent and convective motions accomplish the vertical transports of heat and moisture that is supplied by surface sensible and latent heat fluxes, respectively.

Here we use the Vial et al. (2016) framework described in Sect. 2.2 to better interpret the contrasting model behaviors described in the preceding sections and provide a broader perspective:

- In a warming climate, all numerical models (LES, GCM, SCM) tend to simulate a more vigorous convective mixing due to increased latent heat flux (e.g., Rieck et al. 2012; Blossey et al. 2013; Vogel et al. 2016; Brient et al. 2015; Vial et al. 2016), yielding ${\mathrm {d}}\mu > 0$ in Eq. 6. But this is not necessarily associated with changes in cloud fraction. For a given latent heat flux, the efficiency of the convective mixing at desiccating low-level clouds (i.e., ${\mathcal {C}}$) depends on where the convective drying maximizes with respect to the cloud layer (Vial et al. 2016). In most LES and according to the cumulus-valve mechanism, the convective mass flux originates in the subcloud layer where there is no cloud to desiccate. So ${\mathcal {C}}$ is likely to be very small. However, this appears to be different in LES on larger domains, when convection organizes into more vigorous and deeper clusters. It has recently been shown that some larger convective plumes may originate within the cloud layer, and that these convective fluxes alone could dry the lower troposphere, just above cloud-base (Neggers 2015b; Seifert et al. 2015). Therefore, in larger domain simulations ${\mathcal {C}}$ might be stronger. On the other hand, in GCM and SCM, ${\mathcal {C}}$ is likely to depend on how the bulk convective mass flux is formulated, as shown in Vial et al. (2016).
- In all models, increased latent heat flux moistens the lower troposphere through turbulent diffusion and convective transport and favors cloud formation, yielding ${\mathcal {T}} > 0$.
- In all the models considered, $\lambda$ (the change in surface evaporation per unit change in the convective mixing) is positive: more convective mixing dries the subcloud layer and increases surface evaporation. However, there might be some disagreement on how effective this is, as this depends on how efficiently increased convection brings dry air to the surface, and to what extent it is accompanied by increased warming. This effect would influence low-level stability and eventually the cloud response to warming (Brient et al. 2015).
- In both Rieck et al. (2012) and Vogel et al. (2016), the LES experimental setup prescribes uniform radiative cooling, and therefore the interaction between latent heat flux and cloud radiative cooling is neglected in these studies (i.e., $\lambda _{\mathrm {r}} = 0$). The coupling between cloud radiative forcing and latent heat flux has been recently identified in Vial et al. (2016) in one model; its robustness across models and in observations remains to be shown.

## Observational Support for Trade-Wind Shallow Cumulus Cloud Feedbacks

As discussed in the previous sections, the primary source of uncertainties in trade-wind cloud responses to warming in numerical models relies in how strong subgrid-scale vertical transports of heat and moisture affect cloudiness near cloud-base in response to changes in the large-scale environment. To this end, numerical experiments made it possible considerable progress on the understanding of the key processes that couple convection, turbulence and cloudiness in trade-wind boundary layers. In addition, several studies have used observations to test our physical understanding on a wide range of timescales and to constrain uncertainties of the simulated cloud changes in a warming climate (e.g., Clement et al. 2009; Qu et al. 2014; Brient and Schneider 2016). However, our confidence in low-level cloud feedbacks remains fairly low as the primary factors controlling low-level cloud variability in nature appear to be strongly dependent on the time and/or spatial scales at which the mechanisms are evaluated. Put differently, observational analyses of the factors controlling the trade-wind cloud responses to warming have not yet helped to clarify the inconsistency of the results that we have found between large-scale numerical models (e.g., GCM and SCM) and high-resolution simulations (e.g., LES).

On large domains and long timescales (interannual, decadal or climatological timescales), sea surface temperature explains a large part of the subtropical low-level cloud variability (e.g., Clement et al. 2009; Qu et al. 2014, 2015; Brient and Schneider 2016), with a reduced cloud cover when the sea surface is warmer. Note, however, that these observational studies consider both stratiform and cumuliform types of low-level clouds. Nevertheless, this present-day relationship appears to be consistent with the lower-tropospheric mixing mechanism that controls the low-level cloud response to a warming climate (Sect. 2.2) in GCM and SCM simulations, as the cloud sensitivity to warming was found to be correlated with enhanced latent heat flux and vertical gradient of moisture between the boundary layer and the free troposphere (Qu et al. 2015), and both contribute to enhance the mixing of dry free tropospheric air into the boundary layer that leads to the reduction in low-level cloudiness (Rieck et al. 2012; Bretherton et al. 2013; Brient and Bony 2013; Vial et al. 2016). The observational studies cited above suggest that models that simulate a stronger cloud decrease in a warming climate (and thus a stronger cloud feedback) are more consistent with observations than models that simulate a weaker cloud feedback, and thus that high climate sensitivities are maybe more credible than low climate sensitivity estimates. This is in line with other studies that have related the low-level cloud feedbacks and/or climate sensitivity estimates with climatological indicators of the present-day lower-tropospheric mixing (such as vertical gradients in temperature and relative humidity, large-scale vertical velocity and shallowness of low clouds): models with a stronger lower-tropospheric mixing in the present-day climate are more efficient in depleting boundary-layer moisture as the climate warms, yielding a stronger low-cloud feedback and ECS ; these models tend to be more consistent with observations than models that simulate a weaker lower-tropospheric mixing in the present-day climate (Sherwood et al. 2014; Brient et al. 2015).

Measurements from the Barbados Cloud Observatory—a facility established on a windward promontory on Barbados to study factors controlling cloudiness in the trades (Stevens et al. 2016)—suggest that models can represent a fairly realistic climatology of the lower-tropospheric trade-wind layer on long-term means but through unrealistic variability on shorter timescales. Analysis of the Barbados data indicates that about 60% of observed cloud variance near cloud-base occurs on timescales smaller than a day (Nuijens et al. 2014, 2015a, b). These data suggest that cloudiness near cloud-base is more controlled by internal feedback processes on short timescales and is relatively independent of large-scale environmental factors (such as subsidence, surface temperature and vertical gradient of humidity) that vary on longer timescales (Bellon and Stevens 2013), consistent with the cumulus-valve mechanism (Sect. 3) and LES results on small domains (Sect. 4.1.1).

In their evaluation of climate model output as compared to the Barbados data, Nuijens et al. (2015b) argue that climate models (1) lack this cloud-base regulation mechanism associated with turbulence and convection that appears to be important in nature on sub-daily timescales and (2) are too sensitive to variations of the large-scale environment (lower-tropospheric relative humidity and thermal stratification) on timescales longer than a day (Nuijens et al. 2015a, b). Furthermore, observed cloud fraction at the inversion dominates the total variance in boundary-layer clouds and explains the seasonality of low-level cloudiness, with larger cloud cover (in winter) when surface winds and trade-wind inversions are stronger (Nuijens et al. 2014, 2015b). These relationships are not captured in climate models either. Because the Nuijens et al. studies point to relationships that are relevant for the interpretation of the simulated shallow cumulus cloud feedbacks in climate models, they raise the question of whether or not these models can simulate realistic changes in trade-wind cloudiness in a warming climate. However, one may also question whether these observed relationships capture all the interactions between the trade-wind boundary layer and the larger scale mesoscale organization that might be necessary to interpret the low-level cloud changes on a sufficiently large domain, which is also what matters for the climate sensitivity problem.

This question could be addressed with the planned field campaign $\hbox {EUREC}^4\hbox {A}$ that will take place on January–February 2020 over a large oceanic area east of Barbados (Bony et al. in revision). Featured with its large experimental domain, and by linking profiles of cloudiness to large-scale fluxes of moisture and energy, including estimates of the cumulus mass flux, this campaign will make it possible to quantify macrophysical properties of shallow cumulus clouds as a function of the large-scale environment and thus to assess the existence of the model-based mechanisms that were discussed here under a wide range of large-scale conditions: (1) the vertical distribution of trade-wind cumulus clouds and its relation to convective mixing, latent heat flux and cloud radiative forcing (Vial et al. 2016) the non-uniformity in the spatial distribution of cloud-base area and its impact on the dynamics of trade-wind boundary layers and associated clouds (Neggers 2015b); and (3) the role of organized convection on trade-wind clouds (Seifert et al. 2015; Vogel et al. 2016). More information on the $\hbox {EUREC}^4\hbox {A}$ field campaign and scientific goals are provided in Bony et al. in revision.

## Synthesis

Fair-weather cumulus clouds, covering large areas of the tropical and subtropical oceans in the trade-wind regions, play a central role in the tropical cloud feedback uncertainties in climate models. Climate models predict different low-level cloud changes in response to warming, which results in a large dispersion in model-based estimates of cloud feedback and climate sensitivity. This large dispersion in model responses arises from differences in the balance of the key boundary-layer physical processes that are parameterized in climate models, especially convection and turbulence. Given the importance of low-level cloud feedbacks in climate change projections, understanding the factors controlling the low-level cloudiness across a wide range of temporal and spatial scales in a hierarchy of numerical models and in observations has emerged as an active research area.

Based on a review of past studies on this issue, we have identified three emergent topics for which further investigation would help understand and constrain shallow cumulus cloud feedbacks:

- the vertical distribution of shallow cumulus cloud layers and its relation to convective mixing, surface fluxes and cloud radiative forcing,
- the impact of the probability distribution of cumulus cloud-base areas on the dynamics of trade-wind boundary layers and associated clouds,
- the role of mesoscale organization, and accompanying episodes of deeper convection, in the trade cumulus variability and feedbacks.

These emergent topics would strongly benefit from combined analyses of high-resolution modeling and field experiments on large domains such as those discussed in this review.
